# MedScanGAN: Synthetic PET & CT Scan Generation Using Conditional Generative Adversarial Networks for Medical AI Data Augmentation

**DOI:** 10.3390/bioengineering13030281

**Published:** 2026-02-27

**Authors:** Agorastos-Dimitrios Samaras, Ioannis D. Apostolopoulos, Nikolaos Papandrianos

**Affiliations:** 1 Department of Energy Systems, University of Thessaly, 413 34 Larisa, Greece; agsamaras@uth.gr; 2 Department of Energy Systems, Artificial Intelligence, Computational Methods & Technological Applications (ACTA), University of Thessaly, Gaiopolis Campus, 413 34 Larisa, Greece; iapostolopoulos@uth.gr

**Keywords:** synthetic data generation, generative adversarial networks, medical imaging, PET/CT, Non-Small-Cell Lung Cancer, data augmentation, computer-aided diagnosis, deep learning

## Abstract

This study tackles the challenge of data scarcity in medical AI, focusing on Non-Small-Cell Lung Cancer (NSCLC) diagnosis from Positron Emission Tomography (PET) and Computed Tomography (CT) images. We introduce **MedScanGAN**, a conditional Generative Adversarial Network designed to generate high-fidelity synthetic PET and CT images of Solitary Pulmonary Nodules (SPNs) to enhance computer-aided diagnosis systems. The framework incorporates advanced architectural features, including residual blocks, spectral normalization, and stabilized training strategies. MedScanGAN produces realistic images—particularly for PET representations—capable of plausibly misleading medical professionals. More importantly, when used to augment training datasets for established deep learning models such as YOLOv8, VGG-16, ResNet, and MobileNet, the synthetic data significantly improves NSCLC classification performance. Accuracy gains of up to **+5.8** absolute percentage points were observed, with YOLOv8 achieving the best results at **94.14% accuracy**, **93.12% specificity**, and **95.33% sensitivity** using the augmented dataset. The conditional generation mechanism enables the targeted synthesis of underrepresented classes, effectively addressing class imbalance. Overall, this work demonstrates both state-of-the-art medical image synthesis and its practical value in improving real-world diagnostic systems, bridging generative AI research and clinical pulmonary oncology.

## 1. Introduction

The diagnostic challenges associated with NSCLC, the most prevalent form of lung cancer often diagnosed at advanced stages, have driven significant research interest in computer-aided classification systems. Previous work has established the efficacy of deep learning approaches, with YOLOv8 emerging as the optimal architecture, achieving 92.3% accuracy in classifying benign versus malignant SPNs using PET and CT imaging [1]. However, these systems face fundamental limitations rooted in medical data scarcity, patient privacy concerns, annotation costs by medical experts, and class imbalance issues—particularly the critical shortage of malignant cases for training robust diagnostic models.

The generation of synthetic medical images has emerged as a promising solution to address data scarcity in medical AI, with several notable approaches demonstrating varying degrees of success. Early pioneering work by Goodfellow et al. [2] established the fundamental GAN framework that enabled realistic image generation, while Arjovsky et al. [3] introduced Wasserstein GANs that significantly improved training stability for complex datasets. In the medical domain, Yang et al. [4] developed cross-modal medical image synthesis, demonstrating improved performance in multi-modal registration tasks. Zhao et al. [5] introduced a diffusion-based approach for MRI synthesis that showed promising results for data augmentation in neuroimaging applications. For pulmonary imaging specifically, Shah et al. [6] explored GAN-based data augmentation for COVID-19 detection in chest X-rays, achieving modest improvements in classification accuracy. Researchers in [7] developed a conditional GAN framework for lung nodule synthesis in CT images, though their approach was limited to single-modality applications. More recently, Huang et al. [8] proposed a federated learning approach with synthetic data generation for multi-institutional collaborations in lung cancer screening. Additionally, research work in [8] investigated style-based GAN architectures for high-resolution medical image generation, achieving impressive visual quality but with limited validation on downstream diagnostic tasks. Clinically, GAN-based augmentation has been shown to yield improvements in diagnostic model performance. Synthetic CT liver lesion images improve sensitivity and specificity of CNN classifiers compared to traditional augmentation alone, underscoring the practical impact of generative data on medical AI performance [9,10,11]. These findings are echoed in systematic reviews that confirm the broad relevance of GANs for medical image augmentation, highlighting applications in areas such as oncology, cardiology, ophthalmology and lung imaging where synthetic samples help address label scarcity and enhance model training [12].

Nevertheless, despite the plethora of contemporary studies, almost all approaches face significant limitations: they typically focus on single modalities, lack integration with established diagnostic pipelines, provide limited validation of actual diagnostic performance improvements, and often do not address the critical challenge of maintaining explainable AI compatibility when using synthetic data for augmentation. What fundamentally differentiates the present work, is the comprehensive framework specifically designed for NSCLC diagnosis that not only generates high-quality PET and CT representations but also demonstrates measurable performance enhancements in established diagnostic models (YOLOv8, VGG-16, ResNet, MobileNet), while also maintaining compatibility with existing explainable AI mechanisms. Our work provides an integrated solution that validates both the quality of generated images and their practical utility in improving real-world diagnostic accuracy, particularly addressing the class imbalance problem through targeted conditional generation of underrepresented malignant cases. Traditional data augmentation techniques, while beneficial, provide limited diversity and may fail to capture the full range of clinically relevant variations needed for training generalized medical AI systems, motivating the use of more advanced generative approaches [10,13]. The opacity of these AI models further complicates clinical adoption, necessitating enhanced explainability and trust-building mechanisms. Prior research demonstrated the potential of deep learning in NSCLC diagnosis while highlighting the need for larger, more diverse datasets to improve model generalization and reliability.

In the current work, we present MedScanGAN, a comprehensive synthetic data generation framework that directly addresses these challenges by producing high-quality PET and CT representations specifically designed to enhance existing NSCLC diagnostic systems. This study’s contributions are multifaceted:

(i) Advanced architecture supporting both PET and CT representation generation with modality-specific optimizations tailored to pulmonary nodule characteristics.
(ii) Improved training methodology incorporating WGAN-GP loss, spectral normalization, and gradient penalty for enhanced stability in medical image generation.
(iii) Targeted conditional generation based on diagnostic labels (benign/malignant) specifically designed to address class imbalance in NSCLC datasets.
(iv) Comprehensive validation framework integrating both quantitative metrics (SSIM, PSNR, FID) and practical utility assessment through existing diagnostic architectures.
(v) Demonstration of significant performance improvements in established NSCLC classification systems when augmented with synthetic data, with PET representations providing measurable enhancements to YOLOv8, VGG-16, ResNet, and MobileNet performance.
(vi) Clinical relevance validation through integration with previously validated explainable AI mechanisms, ensuring maintained interpretability while improving diagnostic accuracy.

This research tries to bridge a critical gap between synthetic data generation research and practical clinical application. Hence, it aims to provide a validated pathway for enhancing medical AI systems, without compromising patient privacy or requiring extensive new data collection efforts.

The remainder of this manuscript is structured as follows. Section 2 (Methods) details the experimental workflow, including the composition of the image dataset, the generative GAN architectures employed, the DL models considered, the rationale behind incorporating GENAI-generated images for model retraining, and the metrics used to assess performance. Section 3 (Results) then reports the outcomes of these experiments, presenting both the performance of the GAN models and the behavior of the retrained DL classifiers when augmented with synthetic images. In Section 4 (Discussion), we analyze and compare the strengths, weaknesses, and broader implications of the proposed approaches in the context of existing literature. Finally, Section 5 (Summary) provides concluding remarks, highlights key insights, and outlines potential avenues for future investigation.

## 2. Materials and Methods

The methodology of this research was executed in a Linux environment, specifically on a system equipped with a 14-core i5 CPU, 32GB DDR4 RAM, RTX-3060 GPU and running Ubuntu 20.04LTS. Python 3.10.12 served as the primary programming language for the project, complemented by various machine learning-specific libraries such as sklearn and torch. The training of the GAN architectures was implemented on the Graphical Processing Unit (GPU), utilizing its CUDA cores. Particular training details for the system are presented in Appendix A.

### 2.1. Experimental Pipeline and Methodology

**Figure 1:**
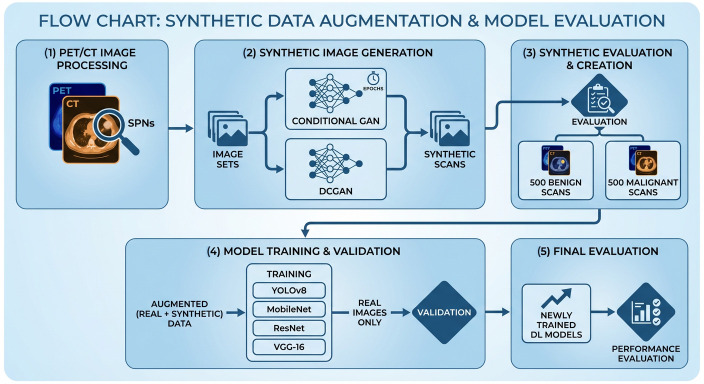
Study’s high-level design. (Source: Created by the author).

The comprehensive evaluation of synthetic data utility for NSCLC diagnosis followed a systematic four-stage pipeline designed to rigorously assess both generative quality and diagnostic enhancement. This structured approach included methodical validation at each phase while maintaining clinical relevance throughout the experimental process. Figure 1 is a high-level flow chart of all the stages of the current project.

**Stage 1: Generative Model Training and Optimization**

The initial phase focused on developing high-quality synthetic image generators through extensive training and evaluation of multiple GAN architectures. The chosen DCGAN and Conditional GAN configurations both underwent thorough optimization using the training methodology with WGAN-GP loss, spectral normalization, and gradient penalty enforcement. Models were trained for 500–1000 epochs with systematic evaluation at regular intervals using quantitative metrics including SSIM, PSNR, and Fréchet Inception Distance (FID). This phase identified optimal model checkpoints based on maximum SSIM scores while ensuring anatomical plausibility through qualitative assessment by medical experts.

**Stage 2: Synthetic Data Generation and Curation**

Following generative model optimization, controlled batches of synthetic PET and CT representations were produced using the best-performing architectures. The generation process employed conditional sampling to maintain balanced class distributions to address dataset imbalance. This curation process resulted in comprehensive synthetic datasets for both PET and CT modalities.

**Stage 3: Diagnostic Model Retraining with Augmented Data**

The core experimental phase involved retraining established NSCLC diagnostic models—YOLOv8, VGG-16, ResNet, and MobileNet—using augmented datasets combining original real images with qualified synthetic samples. Crucially, the training methodology maintained identical architectures and hyperparameter configurations from the original models to ensure fair comparison. The retraining process employed the same optimization strategies and validation protocols as the original study, with synthetic data integrated following carefully designed mixing ratios to maximize beneficial effects while preventing domain shift or quality degradation.

**Stage 4: Performance Evaluation on Real Data**

The last development stage involved evaluation of the augmented models using exclusively real medical images from the held-out test set. This strict validation protocol ensured that performance improvements reflected genuine diagnostic capability enhancement rather than synthetic data memorization or domain adaptation. Models were evaluated using the established diagnostic metrics—accuracy, sensitivity, specificity, ROC-AUC, and confusion matrices—with direct comparison against baseline performances from the original study.

**Stage 5: Performance Evaluation using Expert Opinion**

The final stage of the current work included evaluation of the augmented models utilizing two cohorts of randomized synthetic and real images. By randomly combining 80 real representations and 80 generated, a test image-set was created for PET and another for CT case. This set was passed to an experienced (over 30 years of experience) Nuclear Medicine doctor, who were then asked to guess which images were real and which generated. This step was employed in order to measure the MedScanGAN’s image generation quality capabilities from a human perspective. It is highly important to note, that the main purpose of the current work is to create generated image-sets, that are able to enhance ML/DL models for malignant/benign classification tasks and circumvent the data scarcity issue many similar medical applications face; it is not to create realistic enough images to be able to mislead human interactors. Nevertheless, this stage is useful as a general indication of the generated image quality.

### 2.2. Image Set—Patient Population

This study utilized a comprehensive dataset comprising 456 participants who underwent hybrid PET/CT imaging for SPN characterization. The cohort demonstrated a well-balanced distribution of diagnostic outcomes, with 234 individuals (51.32%) receiving malignant SPN diagnoses confirmed by medical experts, while the remaining 222 participants (48.68%) were classified as benign cases. The patient population was predominantly male (70.37%), with ages ranging from 46 to 89 years and a mean age of 67 years. Body composition varied substantially across participants, as evidenced by Body Mass Index (BMI) values spanning from 14.36 to 40.88, encompassing categories from underweight to severe obesity according to World Health Organization classification standards [14].

All imaging was performed using a GE Healthcare IQ3 hybrid PET/CT scanner, which incorporates three detector rings with a 15 cm field of view to reconstruct 35 axial images at 4.25 mm intervals. Comprehensive three-dimensional volumetric data were acquired through multiple bed positions with patients in the supine position. The CT component employed sixteen detectors of 3.75 mm size with 1.5 pitch and 5 mm collimation, utilizing exposure parameters of 120–140 kVp and 80 mAs in free-breathing acquisition mode. The PET imaging involved administration of fluorodeoxyglucose (FDG) radiotracer, which accumulates in metabolically active tissues, enabling the detection of gamma rays emitted from positron-electron interactions to construct detailed metabolic activity maps [15]. Concurrently, the CT component provided high-resolution anatomical information through X-ray cross-sectional imaging, crucial for precise anatomical localization and characterization of internal structures [16].

Medical interpretation integrated both modalities, with physicians and radiologists conducting cross-examinations of PET metabolic data, high-resolution CT images (CT1.25), and quantitative biomarkers including SUVmax measurements to establish definitive diagnoses. The study excluded representations without identifiable solitary pulmonary nodules and cases featuring lung nodules exceeding 3 cm in diameter. Data collection occurred at the Clinical Sector of the Department of Nuclear Medicine, University Hospital of Patras, between February 2019 and February 2022, with ethical approval granted by the Institutional Ethical and Research Committee (protocol 108/10-3-2022). The retrospective nature of the study permitted waiver of individual informed consent, while stringent anonymization protocols ensured patient confidentiality in accordance with Helsinki Declaration principles.

The imaging dataset was systematically organized into two primary modalities: PET and CT representations. Each modality was subsequently partitioned into three distinct subsets following conventional machine learning practices, with 70% allocated for training, 20% for validation, and 10% for testing. This partitioning strategy enabled model development and parameter calibration using the training set, performance optimization via the validation set, and final evaluation using the held-out test set. Prior to model development, all images underwent standardized preprocessing, including the addition of diagnostic label layers (benign/malignant) and resolution adjustment tailored to specific application requirements. This work extends previous research efforts [1] that focused exclusively on real clinical data. The image set is now synthetically expanded with artificially generated medical images.

### 2.3. GAN Architectures

This study implemented and evaluated two distinct generative adversarial network architectures specifically optimized for medical image synthesis of PET and CT representations. The architectural selection was designed to provide both baseline performance assessment and conditional generation capabilities, enabling comprehensive evaluation of synthetic data quality and its utility for enhancing NSCLC diagnostic systems. Both implementations incorporated advanced training stabilization techniques to address the unique challenges of medical image generation, including anatomical consistency preservation and diagnostic feature maintenance.

**Figure 2:**
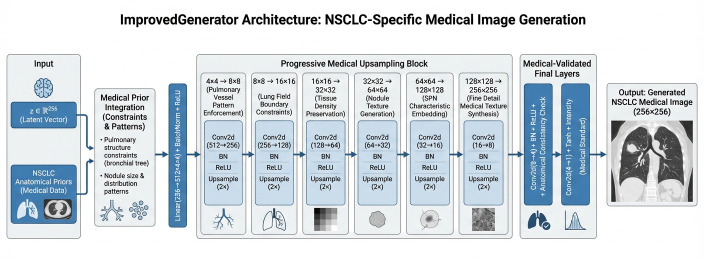
Improved Generator layer’s high-level design. (Source: Created by the author).

**Figure 3:**
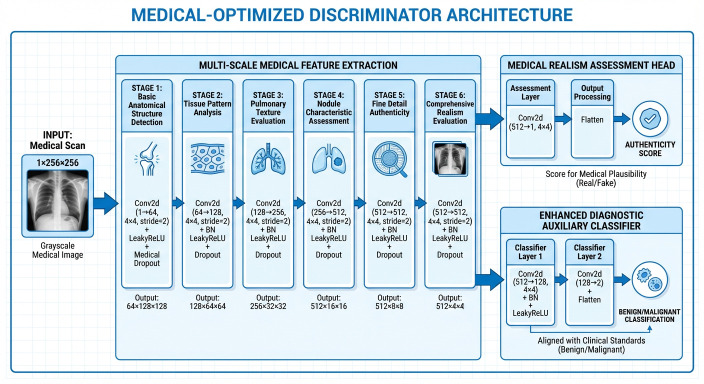
Improved Discriminator layer’s high-level design. (Source: Created by the author).

The DCGAN architecture implemented in this study builds upon the foundational work of Radford et al. [17], incorporating convolutional neural networks into both generator and discriminator components, as depicted in Figure 2 and Figure 3. Our implementation features a generator with progressive upsampling blocks that transform latent vectors of dimension 256 through a series of transposed convolutions, batch normalization, and ReLU activations to produce 256 × 256 medical images. The discriminator employs strided convolutional layers with batch normalization and dropout for robust feature extraction and classification. This architecture’s stability and relatively simple design made it well-suited for our medical imaging context, providing a strong baseline for synthetic PET and CT representation generation. The DCGAN implementation demonstrated particular effectiveness in capturing global anatomical structures while maintaining training stability across extended training sessions.

The Conditional GAN architecture implemented in this work extends the DCGAN framework by incorporating diagnostic labels (benign/malignant) to guide the generation process, following the principles established by Mirza and Osindero [18]. Our cGAN implementation features an enhanced label embedding system that projects categorical labels into the latent space through a multi-layer projection network, and then concatenates these embeddings with noise vectors before the initial generator projection. This conditioning mechanism enables the targeted synthesis of pathology-specific medical images, allowing for controlled generation of either benign or malignant pulmonary nodule characteristics. The discriminator includes an auxiliary classifier that simultaneously evaluates image authenticity and predicted diagnosis, creating a dual learning objective that improves both image quality and diagnostic relevance. This architecture proved particularly valuable for addressing class imbalance in our NSCLC dataset by enabling selective augmentation of underrepresented malignant cases.

Both architectures were trained using an improved methodology incorporating Wasserstein GAN with Gradient Penalty (WGAN-GP) optimization, drawing from the stability enhancements proposed by Gulrajani et al. [19]. Our implementation included spectral normalization, customized learning rates (1 × 10^−4^ for generators, 4 × 10^−4^ for discriminators), and gradient penalty enforcement (λ = 10) to maintain training stability across 500–1000 epoch training sessions. The enhanced training framework demonstrated significant performance improvements over standard training approaches, with DCGAN achieving 50% SSIM improvement and Conditional GAN showing 16% improvement on CT representations, while both architectures achieved exceptional performance on PET representations with SSIM values exceeding 0.69.

Quantitative SUV (Standardized Uptake Value) metrics were not preserved or evaluated in the current study. The generated PET images underwent normalization to [0, 1] during preprocessing and training, which removes the original quantitative SUV scale. As such, the generated images are suitable for qualitative visual assessment and structural pattern recognition but not for quantitative analysis requiring accurate SUV values.

### 2.4. DL Models

The present investigation employed four established deep learning architectures for medical image classification: YOLOv8 (You Only Look Once), VGG-16 (Visual Geometry Group 16-layer), ResNet (Residual Network), and MobileNet. This selection represents a spectrum of proven computational approaches with demonstrated efficacy across diverse image classification domains [20,21,22,23,24,25,26,27].

The YOLOv8 [28] framework implements an efficient object detection methodology characterized by single-pass processing that simultaneously predicts bounding coordinates and class probabilities. Originally conceptualized by Redmon and colleagues, this architecture has evolved through successive iterations to achieve enhanced performance capabilities. In medical imaging contexts, YOLO’s rapid processing characteristics enable timely identification and spatial localization of pathological findings, making it particularly suitable for clinical scenarios requiring expedited diagnostic support. The unified architectural approach facilitates robust anomaly detection across varied medical imaging modalities.

VGG-16 [29] constitutes a deep convolutional neural network configuration developed by the Visual Geometry Group at Oxford University. This architecture employs a sequential arrangement of convolutional layers that progressively extract hierarchical feature representations from input imagery. The model’s substantial depth facilitates the capture of nuanced visual patterns, establishing it as a benchmark in computer vision applications. Within medical image analysis, VGG-16s comprehensive feature learning capacity enables discrimination of subtle pathological manifestations that may evade detection in shallower network architectures.

ResNet [30] introduces innovative residual learning mechanisms through skip connections that mitigate gradient dissipation challenges in deep network training. This architectural strategy, pioneered by He and collaborators, enables effective training of substantially deeper networks than previously feasible. For medical imaging applications, ResNet’s enhanced depth facilitates identification of complex diagnostic patterns, particularly in oncological imaging where subtle morphological variations require sophisticated analytical capabilities. The residual learning paradigm has demonstrated particular utility in tumor characterization tasks across multiple imaging modalities.

MobileNet [31] represents a computationally optimized architecture employing depth-wise separable convolutions to maintain classification accuracy while minimizing computational overhead. Developed by Google research scientists, this framework achieves an effective balance between performance efficiency and resource requirements. In medical contexts, MobileNet’s streamlined design enables deployment in resource-constrained environments, including mobile diagnostic platforms and point-of-care imaging systems. The architecture’s adaptability supports practical implementation across diverse healthcare settings without compromising diagnostic reliability, thereby expanding accessibility to advanced medical image analysis capabilities.

### 2.5. Results Evaluation

The study employed a multi-faceted evaluation approach encompassing both synthetic image quality assessment and diagnostic performance validation. The evaluation framework was designed to comprehensively assess the fidelity of generated medical images and their practical utility in enhancing NSCLC diagnostic systems.

#### 2.5.1. Synthetic Image Quality Metrics

SSIM [32] measured the perceptual quality and structural preservation between generated and real medical images, assessing luminance, contrast, and structural similarity. This metric is crucial for medical imaging as it evaluates the preservation of anatomical structures and pathological features essential for accurate diagnosis. The SSIM values range from −1 to 1, with higher values indicating better structural preservation.

PSNR quantified the reconstruction quality by measuring the ratio between the maximum possible power of an image and the power of corrupting noise. Expressed in decibels (dB), higher PSNR values indicate better image quality with fewer artifacts, which is vital for maintaining diagnostic integrity in synthetic medical images.

FID [33] assessed the distributional similarity between real and generated images by comparing feature representations extracted from a pre-trained Inception network. Lower FID scores indicate better alignment between synthetic and real image distributions, ensuring that generated samples capture the essential characteristics of authentic medical representations.

Mean Squared Error (MSE) [34] provided pixel-level accuracy assessment by computing the average squared difference between corresponding pixels in real and generated images. While less perceptually relevant than SSIM, MSE offers complementary information about overall image fidelity.

SSIM, PSNR, and MSE are full-reference (pairwise) metrics that require a corresponding ground-truth image. Since our GAN operates in a diagnosis-conditional but not pixel-paired setting, there is no natural one-to-one correspondence between generated and real images. To enable the use of these metrics, we defined an explicit matching protocol: for each generated image, a reference real image was randomly selected from the held-out test set belonging to the same imaging modality and diagnosis category (benign or malignant). SSIM, PSNR, and MSE were then computed between the generated image and this randomly assigned reference. We acknowledge that this pairing strategy does not reflect reconstruction fidelity in the strict sense, but rather provides a class-consistent structural and pixel-level similarity assessment. Empirically, incorporating this evaluation-driven filtering mechanism during model development contributed to improved overall performance of MedScanGAN.

#### 2.5.2. Diagnostic Performance Metrics

Following synthetic data augmentation, the enhanced diagnostic models were evaluated using established classification metrics to quantify improvements in NSCLC detection.

Accuracy measured the overall correctness of the classification model, calculated as:

(1) Accuracy = (TP + TN)/(TP + TN + FP + FN)

where TP represents True Positives, TN represents True Negatives, FP represents False Positives, and FN represents False Negatives.

Sensitivity (Recall) assessed the model’s ability to correctly identify malignant cases, crucial for early cancer detection:

(2) Sensitivity = TP/(TP + FN)

Specificity evaluated the model’s capability to correctly identify benign cases, reducing unnecessary interventions:

(3) Specificity = TN/(TN + FP)

Receiver Operating Characteristics (ROC) analysis provided comprehensive performance visualization across different classification thresholds, with the Area Under the Curve (AUC) quantifying overall diagnostic capability. The ROC curve plots true positive rate against false positive rate, enabling optimal threshold selection for clinical deployment [35].

Confusion matrices offered detailed breakdowns of classification performance, revealing patterns in misclassification and highlighting specific strengths and weaknesses of each augmented model.

## 3. Results

The result section is organized into two parts. First, the results of generative medical image creation are presented. Metrics show how each architecture performed and which one produced the better results. The Section 2, is indicative of the overall goal of the current study. It showcases how DL models can be readily enhanced by the usage of realistic synthetic image sets.

### 3.1. GAN Architectures Results

The evaluation of generative adversarial network architectures demonstrated promising performance in synthetic medical image generation, with particularly outstanding results achieved for PET representation synthesis. Both DCGAN and Conditional GAN architectures produced clinically relevant synthetic images, though significant modality-dependent performance variations were observed.

**Figure 4:**
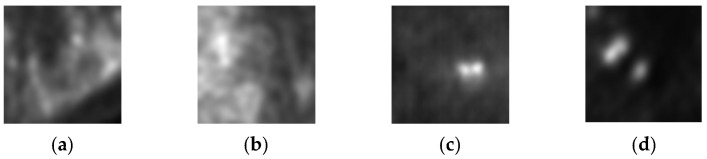
PET cases: (**a**) real benign, (**b**) generated benign, (**c**) real malignant, (**d**) generated malignant.

As summarized in Table 1, both generative architectures achieved good success in PET representation synthesis, with near-identical SSIM values of approximately 0.694. The Conditional GAN architecture attained a peak SSIM of 0.6947 with a PSNR of 9.49 dB after 450 training epochs, while the DCGAN configuration reached a comparable SSIM of 0.6941 with a superior PSNR of 10.06 dB after 500 epochs. These results represent valuable performance in medical image synthesis, with SSIM values approaching 0.7 indicating high structural fidelity preservation—a critical requirement for maintaining diagnostic utility in synthetic PET representations. The consistently high performance across both architectures suggests that PET imaging characteristics, particularly the metabolic activity patterns and smoother texture distributions, are well-suited to GAN-based synthesis approaches. Examples of AI generated PET representations are depicted in Figure 4, along with real counterparts.

Generated PET images showcased remarkable similarity to real representations, from the human perspective, as well. The human expert classified correctly 59 out of 160 images (36.875 accuracy) as real/generated.

**Figure 5:**
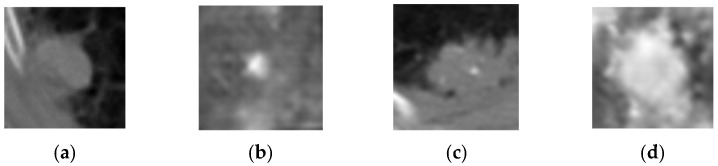
CT cases: (**a**) real benign, (**b**) generated benign, (**c**) real malignant, (**d**) generated malignant.

CT representation generation presented greater challenges, as evidenced by the results in Table 2. The DCGAN architecture achieved the highest CT performance with an SSIM of 0.4198 and PSNR of 7.18 dB after 350 training epochs, while the Conditional GAN reached an SSIM of 0.3851 and PSNR of 6.83 dB after extended training of 950 epochs. The significantly worse performance levels for CT synthesis reflect the inherent complexity of anatomical structure generation compared to metabolic pattern synthesis in PET imaging. The approximately 40% lower SSIM values for CT compared to PET highlight the fundamental differences in modality characteristics, with CT’s detailed anatomical textures, bone structures, and tissue density variations potentially presenting greater challenges for generative modeling. In similar fashion of AI generated CT representations are depicted in Figure 5, along with real counterparts.

Contrary to the PET case, generated CT images did not showcase the same affinity in misleading medical experts. The human expert classified correctly 135 out of 160 images (84.37% accuracy) as real/generated, a figure notably higher than the PET counterpart.

### 3.2. Data Augmentation Results

The integration of synthetic PET representations into the training pipeline demonstrated significant improvements across all diagnostic architectures, with particularly notable gains in sensitivity and overall model robustness. The augmentation strategy, which leveraged the synthetic PET images generated by both Conditional GAN and DCGAN architectures, resulted in measurable performance enhancements.

#### 3.2.1. PET Image Set

As detailed in Table 3, the baseline performance of diagnostic models on PET images again established YOLOv8 as the leading architecture with 94.14% accuracy. All models showcased significant increases in their respective metrics. The biggest improvement was noticed on the MobileNet. Following synthetic data augmentation, the performance landscape evolved very positively.

#### 3.2.2. CT Image Set

Similarly, Table 4 illustrates the accuracy, sensitivity, and specificity outcomes derived from applying the models on the validation image set of CT medical representations. Once again, the YOLOv8-derived model demonstrated superior performance, achieving the highest scores for accuracy and specificity. This time, however, the increase in performance was smaller than the PET modality.

## 4. Discussion

The current study has multiple goals. First, it explores the possibility of producing high-quality, realistic synthetic PET & CT representations. For this, multiple architectures with different generator/discriminator combinations were employed and measured. Furthermore, the eventual scope of the present work was to use batches of synthetic image data to train established DL architectures and measure their effect. This was meant to tackle multiple issues on the aspect of medical image sets currently faced by the research community.

Indeed, there was a remarkable performance for both GAN architectures on PET representation generation (SSIM > 0.69), whereas a somewhat more mediocre performance on CT representations (SSIM ~0.40), as depicted in Table 1 and Table 2. This difference highlights fundamental differences in modality characteristics that directly impact generative modeling efficacy. PET imaging, with its functional representation of metabolic activity and smoother texture distributions, appears inherently more amenable to GAN-based synthesis. The metabolic patterns in PET representations apparently follow more predictable statistical distributions that the generative models employed could effectively capture and reproduce. Conversely, CT imaging presented greater challenges, possibly due to its complex anatomical structures, tissue density variations, and intricate textures that require more sophisticated spatial reasoning capabilities.

While the generated CT images demonstrate sufficient quality to serve as effective data augmentation for DL and ML model training, they fall short of the fidelity required to deceive experienced medical professionals. In stark contrast, the generated PET images exhibit a dual advantage: they not only provide substantial augmentation benefits during model training, but also achieve a level of realism that is sufficient to potentially mislead even trained specialists. The reason is that CT representations are much more complex than their PET counterparts, depicting anatomical information. On the other hand, PET representations are possibly easier to generate by means of AI, as the main information they convey (FDG uptake) is mainly represented by change in brightness on hotspots.

This differential quality between modalities highlights the varying challenges in generating convincing medical images across different imaging types. Ergo, PET representation generation may represent a more mature application of generative adversarial networks in medical imaging. Nevertheless, the generation of synthetic images that are realistic enough to mislead human professionals is out of the scope of the current study.

The high-quality PET synthesis suggests that metabolic imaging may be particularly well-suited for synthetic data augmentation in oncology applications, where metabolic activity patterns are crucial for malignancy characterization. The less high-performant CT part, while still clinically valuable, indicates that anatomical imaging may require additional architectural innovations or hybrid approaches to achieve similar quality levels. Perhaps, a wider image set for training could also help mitigate these prohibiting factors.

The augmentation results reveal interesting patterns in how synthetic data improves diagnostic performance, as showcased by Table 3 and Table 4. The consistent improvements across all architectures for PET-based classification (1.84–4.82 absolute percentage points of accuracy gains) demonstrate the universal applicability of synthetic augmentation, though the magnitude of improvement varies by architecture. MobileNet showcased the largest relative improvement (+5.8), suggesting that lighter architectures with greater susceptibility to overfitting on limited datasets may benefit disproportionately from synthetic augmentation. This finding may have implications for resource-constrained clinical environments where computational efficiency is crucial. The differential impact on sensitivity versus specificity metrics provides insights into how synthetic data influences diagnostic behavior. The maintained or improved sensitivity across all models is clinically significant, as it indicates enhanced capability to identify malignant cases without increasing false negatives—a crucial consideration in oncology where missed diagnoses carry severe consequences. The balanced improvements across metrics suggest that synthetic augmentation enhances overall diagnostic capability rather than simply optimizing for specific metric targets.

The successful integration of synthetic data with existing diagnostic pipelines could indicate a strong advancement toward clinical adoption. Previous approaches may have treated synthetic generation and diagnostic enhancement as separate challenges, but our integrated framework demonstrates that maintained applicability is feasible while improving performance. This is quite important for clinical settings, where model transparency and decision justification are essential for physician trust and regulatory approval.

The modality-specific augmentation efficacy has provided diverse results and insights. PET-based diagnostic systems appeared much more expendable with synthetic data. However, this is also directly affected by the better performance of the GAN architectures on PET image sets. CT-based systems may require more careful implementation, potentially focusing on specific architectures (VGG-16, MobileNet) that showed clear benefits, while monitoring for potential specificity trade-offs observed in some configurations. Nevertheless, the performance of the employed GAN architectures on the CT image set should be taken into consideration. Had there been better results on artificial image synthesis, perhaps the CT-based diagnostic systems would have shown equal improvement.

The observed architecture-specific responses to augmentation suggest that optimal synthetic data integration strategies may need to be tailored to specific model characteristics. Future research should investigate architecture-aware augmentation protocols that maximize benefits while minimizing potential negative interactions. Moreover, the current study focused on NSCLC diagnosis, but the framework’s applicability to other medical domains requires validation.

The successful implementation of synthetic data augmentation addresses several ethical and practical challenges in medical AI. By reducing dependency on large real datasets, our approach mitigates privacy concerns and facilitates research in data-scarce scenarios, such as rare diseases or specialized patient populations. The conditional generation capability specifically addresses equity concerns by enabling balanced representation of underrepresented conditions.

A key strength of the present work lies in the robust and carefully optimized GAN training framework. Specifically, the incorporation of spectral normalization, asymmetric and customized learning rates (1 × 10^−4^ for generators and 4 × 10^−4^ for discriminators), and gradient penalty enforcement (λ = 10) was paramount for maintaining training stability across extended training regimes of 500–1000 epochs. These design choices effectively mitigated common GAN failure modes such as mode collapse and unstable convergence, which are particularly pronounced in high-resolution medical imaging tasks. As a result, the enhanced framework yielded substantial improvements. These findings underscore that architectural choice alone is not always sufficient by itself; rather, training strategy optimization is a critical determinant of generative performance, especially for complex modalities such as CT. The observed improvements directly translated into more effective synthetic augmentation, reinforcing the practical relevance of the proposed framework for real-world diagnostic pipelines.

There are, however, limitations that warrant consideration. The current 2D slice generation, while effective for many diagnostic tasks, may not capture the full 3D contextual information important for comprehensive pulmonary nodule assessment. Future work could explore 3D volume generation to provide more complete anatomical context. The CT generation quality, while sufficient for augmentation purposes, has room for improvement toward diagnostic-level quality. Nevertheless, such approaches may prove too difficult to implement due to the sheer graphical and analytical power required for 3D rendering.

Overall, from a practical perspective, the framework provides a sustainable pathway for continuous improvement of medical AI systems, without requiring extensive new data collection. This is particularly valuable in rapidly evolving medical fields where new imaging protocols or diagnostic criteria emerge frequently. Hopefully, such works can be used as a blueprint for the growth of medical related AI applications and solutions.

## 5. Conclusions

This study presents MedScanGAN, a comprehensive framework for synthetic PET and CT representation generation that demonstrates both exceptional generative performance and significant practical utility in enhancing NSCLC diagnostic systems. Our work establishes that high-quality synthetic medical images can meaningfully improve real-world diagnostic accuracy while maintaining explainable AI compatibility—a crucial consideration for clinical adoption.

The research reveals fundamental differences in generative suitability between medical imaging modalities, with PET representations achieving remarkable synthesis quality compared to CT representations, directly influencing augmentation efficacy. The conditional generation capability successfully addresses class imbalance challenges. Across all evaluated architectures, synthetic data augmentation produced measurable improvements, with particularly strong results for PET-based classification, where accuracy improvements reached 4.82 in absolute percentage points. Ergo, these findings bridge critical gaps between generative AI research and clinical application, providing a validated pathway for enhancing medical AI systems without compromising patient privacy or requiring extensive new data collection.

Possible future work may include extending the framework to 3D volume generation, exploring domain adaptation for other medical specialties, and developing architecture-specific augmentation protocols. The success demonstrated in enhancing NSCLC diagnosis positions synthetic data augmentation as a template methodology in medical AI development, providing potential to accelerate innovation, while addressing fundamental challenges of data scarcity and model generalization.

## Figures and Tables

**Table 1:** PET Representation Generation Performance for NSCLC Enhancement (best results highlighted in bold).

Architecture	SSIM	Training Epochs	Potential
**Conditional GAN**	**0.6947**	450	High
**DCGAN**	0.6941	500	High

**Table 2:** CT Representation Generation Performance for NSCLC Enhancement (best results highlighted in bold).

Architecture	SSIM	Training Epochs	Potential
**Conditional GAN**	0.3851	950	Moderate
**DCGAN**	**0.4198**	350	Moderate

**Table 3:** Metrics scores achieved on the PET image set (best results highlighted in bold).

*Model*	*Dataset*	*Accuracy (%)*	*Specificity (%)*	*Sensitivity (%)*
*YOLOv8*	Original PET	92.30	90.91	93.62
	Augmented PET	**94.14 (+1.84)**	**93.12 (+2.21)**	**95.33 (+1.71)**
*VGG-16*	Original PET	87.91	86.36	89.36
	Augmented PET	**91.16 (+3.25)**	**88.89 (+2.53)**	**93.18 (+3.82)**
*ResNet*	Original PET	85.71	86.36	85.11
	Augmented PET	**89.17 (+3.46)**	**89.55 (+3.19)**	**88.91 (+3.80)**
*MobileNet*	Original PET	83.52	81.82	85.11
	Augmented PET	**88.34 (+4.82)**	**87.11 (+5.29)**	**89.22 (+4.11)**

**Table 4:** Metrics scores achieved on the CT image set (best results highlighted in bold).

*Model*	*Dataset*	*Accuracy (%)*	*Specificity (%)*	*Sensitivity (%)*
*YOLOv8*	Original CT	89.00	97.73	80.85
	Augmented CT	**89.88 (+0.88)**	**96.36 (−1.37)**	**82.77 (+1.92)**
*VGG-16*	Original CT	82.41	79.55	85.11
	Augmented CT	**84.16 (+1.75)**	**80.25 (+0.70)**	**87.43 (+2.32)**
*ResNet*	Original CT	84.62	79.55	89.36
	Augmented CT	**85.29 (+0.67)**	**80.30 (+0.75)**	**89.98 (+0.62)**
*MobileNet*	Original CT	82.42	79.55	85.11
	Augmented CT	**84.42 (+2.00)**	**81.75 (+2.20)**	**87.02 (+1.91)**

## Data Availability

The datasets generated and/or analyzed during the current study are not publicly available due to ethical and legal restrictions associated with the use of anonymized clinical data.

## Appendix A. Training Configuration and Data Generation Details

### Appendix A.1. Training Hyperparameters

The model was trained using the following configuration:

- **Optimizer**: Adam (β_1_ = 0.5, β_2_ = 0.999)
- **Learning rate**: 0.00005
- **Batch size**: 4
- **Number of training epochs**: 500–1000
- **Latent vector dimension**: 100
- **Input image resolution**: 256 × 256 pixels

**Loss Functions:**

- Binary Cross-Entropy (BCE) loss for adversarial training
- Cross-Entropy loss for auxiliary diagnosis classification

### Appendix A.2. Data Augmentation

To improve generalization and reduce overfitting, the following augmentation techniques were applied during training:

• Random horizontal flip with probability *p* = 0.5
• Random rotation within ±5 degrees
• Modality-specific normalization:
  ○ CT images scaled to the range [−1, 1]
  ○ PET images scaled to the range [0, 1]

### Appendix A.3. Synthetic Image Generation and Dataset Mixing

Synthetic images were generated using the best-performing generator checkpoint selected during training.

**Synthetic Data Generation:**

• 1000 synthetic images generated per modality–class combination:
  ○ PET—Benign
  ○ PET—Malignant
  ○ CT—Benign
  ○ CT—Malignant

**Dataset Mixing Strategy:**

• Real-to-synthetic image ratio: 2:1
• Synthetic images were combined with real images prior to training using the specified mixing ratio to augment each modality-class subset.
